# ROCCO: a robust method for detection of open chromatin via convex optimization

**DOI:** 10.1093/bioinformatics/btad725

**Published:** 2023-11-29

**Authors:** Nolan H Hamilton, Terrence S Furey

**Affiliations:** Department of Genetics, University of North Carolina at Chapel Hill, Chapel Hill, NC 27599, USA; Department of Genetics, University of North Carolina at Chapel Hill, Chapel Hill, NC 27599, USA; Department of Biology, University of North Carolina at Chapel Hill, Chapel Hill, NC, 27599, USA

## Abstract

**Motivation:**

Analysis of open chromatin regions across multiple samples from two or more distinct conditions can determine altered gene regulatory patterns associated with biological phenotypes and complex traits. The ATAC-seq assay allows for tractable genome-wide open chromatin profiling of large numbers of samples. Stable, broadly applicable genomic annotations of open chromatin regions are not available. Thus, most studies first identify open regions using peak calling methods for each sample independently. These are then heuristically combined to obtain a consensus peak set. Reconciling sample-specific peak results *post hoc* from larger cohorts is particularly challenging, and informative spatial features specific to open chromatin signals are not leveraged effectively.

**Results:**

We propose a novel method, ROCCO, that determines consensus open chromatin regions across multiple samples simultaneously. ROCCO employs robust summary statistics and solves a constrained optimization problem formulated to account for both enrichment and spatial dependence of open chromatin signal data. We show this formulation admits attractive theoretical and conceptual properties as well as superior empirical performance compared to current methodology.

**Availability and implementation:**

Source code, documentation, and usage demos for ROCCO are available on GitHub at: https://github.com/nolan-h-hamilton/ROCCO. ROCCO can also be installed as a stand-alone binary utility using pip/PyPI.

## 1 Introduction

Nucleosomes, complexes of DNA and histone proteins, comprise the initial stage of chromatin compaction of the genome, reducing its occupying volume and enabling it to fit in cell nuclei (Li and Reinberg 2011). Most nucleosomal DNA is inaccessible for binding by transcription factors (TFs) that regulate gene expression. Genome-wide annotations of non-nucleosomal DNA, or open chromatin, therefore delineate where TFs can readily bind and effectively characterize the current gene regulatory program in a sample. Open chromatin landscapes vary across cell types and conditions, including in disease (Corces and Granja 2018) reflecting cell and condition-specific gene regulation. To better understand this dynamic nature of gene regulation, the identification of open chromatin regions has become an important aspect of molecular studies of complex phenotypes.

Several assays have been developed for genome-wide measurement of open chromatin, including DNase-seq (Boyle *et al.* 2008) and ATAC-seq (Buenrostro *et al.* 2015). These assays generate DNA fragments enriched for open chromatin regions that are then sequenced using short-read sequencers. Resulting reads are aligned to a reference genome, and regions with an enrichment of reads, or “peaks,” are identified as open chromatin. Peak calling is a necessary step as, unlike genes for which annotations are available for many species, there are not comprehensive, predefined standard databases of open chromatin regions.

Studies focused on determining changes in chromatin associated with differing cellular conditions or complex traits normally include many samples. For these studies, it is necessary to define a common set of open chromatin regions, or “consensus peaks,” to facilitate comparisons across sample groups. Typically, consensus peaks are determined by first annotating peaks independently in each sample. Then, these sample-specific peaks are merged based on one of several heuristics including: (i) simply take the maximal set across all samples. This method, though, is particularly vulnerable to anomalous data since peaks from a single sample satisfy the inclusion criterion; (ii) include only peak regions that occur in “all” samples. This is usually too stringent due to variability in data quality across samples, especially when there is an expectation of differences; and (iii) require that peaks be present in at least M=1…K samples, where the boundaries of the consensus peaks allow for some tolerance, *T*, for disparity in nucleotide position. Protocols in the spirit of this general method have been utilized in many open chromatin studies (Bao *et al.* 2015, Wang *et al.* 2018, Bentsen *et al.* 2020, Ming *et al.* 2021). A difficulty in applying such methods is choosing appropriate *M* and *T*—a task manifesting rigid criteria that may ignore some open regions or may include spurious regions and that may not define well-supported peak boundaries.

More statistically sound methods have been developed for handling multiple samples. For the specific case of *K* = 2 samples, the Irreproducible Discovery Rate (Li *et al.* 2011) can be controlled to mitigate calling of irreproducible peaks. However, since most experimental designs include K≫2 samples per group, it is difficult to apply this method broadly. Alternatively, Genrich (available at https://github.com/jsh58/Genrich) offers a method for multiple samples in which *P*-values are combined using Fisher’s method (Fisher 1925). While Genrich has been used successfully in several studies (Hofvander *et al.* 2019, Guerin *et al.* 2021, Salavati *et al.* 2021, Tsaryk *et al.* 2022), the independence assumption of Fisher’s method may be problematic for large numbers of samples and/or in cases involving multiple technical replicates (Roy *et al.* 2019). It also does not explicitly account for specific peak boundaries.

Here, we propose a novel method for identification of open chromatin regions across multiple samples, ROCCO: “Robust Open Chromatin detection via Convex Optimization.” This method offers several favorable features:

- Accounts for both enrichment “and” spatial characteristics of open chromatin signals, the latter of which is an informative but often ignored aspect of ATAC-seq data that can be used to not only better detect regions but also improves on annotating peak boundaries;
- Leverages data from multiple samples without imposing arbitrary “hard” thresholds on a minimum number of samples declaring peaks;
- Is efficient for large numbers of samples;
- Does not require training data or a heuristically determined set of initial candidate regions, which are hard to define given the lack of a priori sets of validated open chromatin regions;
- Employs a mathematically tractable model granting useful performance guarantees.

We formally describe the algorithm utilized by ROCCO for the consensus peak problem and present a theoretical analysis. We then conduct several experiments to investigate ROCCO’s efficacy empirically, using a set of 56 samples from human lymphoblastoid cell lines.

## 2 Materials and Methods

We begin by introducing notation (See Table 1 for a complete notation reference) used throughout this manuscript and describing the structure of signal data used by ROCCO to detect accessible chromatin.

**Table 1.** Notation reference.

Symbol	Description	Default value
*b*	Budget threshold on selected loci	0.035
$c_{1},c_{2},c_{3}$	Weights for score function S(i)	1.0
*f*	Objective function	N/A
*γ*	Fragmentation penalty in Equation (3)	1.0
*K*	Number of input samples	N/A
$\ell_{i}$	Decision variable for *i*th locus	N/A
ℓ	Vector of decision variables	N/A
*L*	Fixed interval size of input signals	50
L	Contiguous genomic region	N/A
*n*	Number of loci in L	N/A
*N*	RR iterations	50
S(·)	Locus score function	N/A
$S_{L}$	*K *×* n* signal matrix over L	N/A
*τ*	Median enrichment threshold in S(·)	0

### 2.1 Notation and definitions

**Figure 1.**
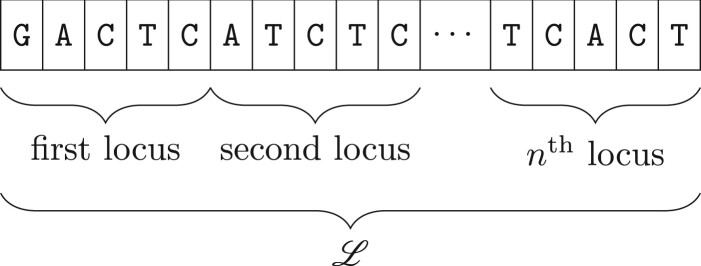
Genomic region L consisting of *n* fixed-width loci containing *L* = 5 nucleotides each.

Let L be a contiguous genomic sequence, e.g. a chromosome, divided into *n* fixed-width loci, each consisting of *L* nucleotides as in Fig. 1. For each sample *j*, we assume access to a signal, $s_{i_{j}}$, computed as a function of observed enrichment based on sequence reads at the *i*th locus. For *K* total samples, this yields a *K *×* n* signal matrix used as input to ROCCO:


(1) $$S_{L}=\left(\begin{array}{cccc} s_{1_{1}} & s_{2_{1}} & \ldots & s_{n_{1}}\\ s_{1_{2}} & s_{2_{2}} & \ldots & s_{n_{2}}\\ \ldots & \ldots & \ldots & \ldots \\ s_{1_{k}} & s_{2_{k}} & \ldots & s_{n_{K}} \end{array}\right)=\left(\begin{array}{cccc} s_{1} & s_{2} & \ldots & s_{n} \end{array}\right),$$

with $s_{i}\in R^{K}$ denoting the column vector of signal values among samples at the *i*th locus. This matrix can be generated with a variety of methods, but a context-specific tool, rocco prep is included as a subcommand in the software implementation for convenience: given a directory of samples’ BAM files, $S_{L}$ is generated with multiple calls to PEPATAC’s (Smith *et al.* 2021) bamSitesToWig.py tool.

### 2.2 Scoring loci

To determine consensus open chromatin regions, we first score each locus while accounting for enrichment (*g*_1_), dispersion among samples (*g*_2_), and a measure of local volatility in enrichment (*g*_3_).

Specifically, we take $g_{1}(i)$ to be the median, and $g_{2}(i)$ to be the median absolute deviation (Pham-Gia and Hung 2001) of the *K* signal values at the *i*th locus:


$$\begin{array}{l} g_{1}(i)=\text{med}\left\{s_{i_{1}},s_{i_{2}},\ldots ,s_{i_{K}}\right\}\\ g_{2}(i)=\text{med}\left\{\left|s_{i_{1}}-g_{1}(i)|,\ldots ,|s_{i_{K}}-g_{1}(i)\right|\right\}. \end{array}$$

Large $g_{1}$ and low $g_{2}$ correspond to regions of high enrichment with little dispersion among samples—a favorable combination of traits to emphasize when predicting accessibility. We also leverage the disparities between enrichment signal values at adjacent loci, normalized by the current locus’s enrichment,


$$\begin{array}{l} g_{3}(i)=\\ \frac{1}{g_{1}(i)+1}\{\begin{array}{cc} |g_{1}(i)-g_{1}(i+1)|, & i=1\\ \max\{|g_{1}(i)-g_{1}(i+1)|,|g_{1}(i)-g_{1}(i-1)|\}, & 1<i<n\\ |g_{1}(i)-g_{1}(i-1)|, & i=n \end{array}. \end{array}$$

**Figure 2.**
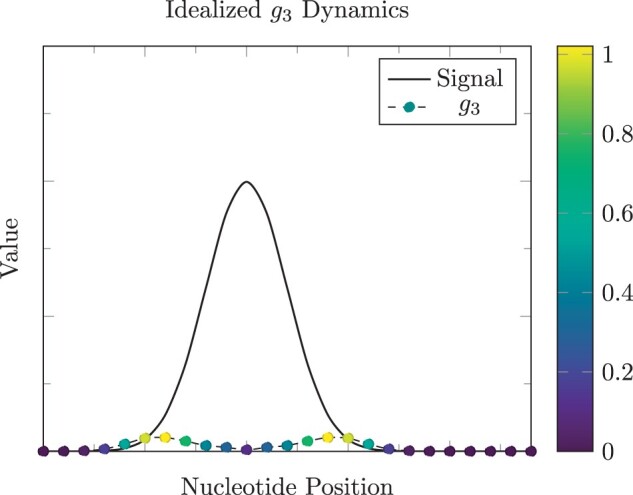
The *g*_3_ term in Equation (2) is designed to more accurately determine peak edges to capture enriched regions in their whole. This function is greatest (yellow marks) near the ends of the enriched region and lowest (dark blue marks) at peak centers in this idealized example.

A fundamental aim of *g*_3_ is to more precisely annotate the edges and immediately adjacent regions of peaks where signals may be low before or after an abrupt shift in enrichment characterizing the nearby peak. See Fig. 2 for a visual demonstration on an idealized, continuous enrichment signal.

In (2), we define the score piece-wise and take a simple linear combination of $g_{1},g_{2},g_{3}$—or set this score to 0 if median enrichment is below a defined threshold. This enrichment threshold can encourage sparsity that may have favorable implications for computational efficiency during optimization and mitigate consideration of regions that are unlikely accessible.


(2) $$S(i)=\{\begin{array}{ll} c_{1}g_{1}(i)-c_{2}g_{2}(i)+c_{3}g_{3}(i) & \text{if}\;g_{1}(i)\geq \tau \\ 0 & \text{if}\;g_{1}(i)<\tau \end{array},$$

where τ≥0 is the minimum enrichment threshold placed on the median signal, and $c_{1},c_{2},c_{3}\geq 0$ are coefficients for each term. By default, each scoring term has the following weights: $c_{1},c_{2},c_{3}=1$, and *τ* = 0. These defaults provide strong performance but can be modified in the software implementation of ROCCO to suit users’ specific needs. For example, users desiring more conservative peak predictions may wish to set τ>0.

S(i)≤0 does not necessarily preclude the corresponding locus from being selected, since the objective function [see Equation (3)] is not completely dependent on the locus score. Note that for default parameters, S(i)≥0.

### 2.3 Optimization

We address open chromatin detection as a constrained optimization problem. Let $\ell \in {\left\{0,1\right\}}^{n}$ be a vector of binary decision variables, where we label $\ell_{i}=1$ if the *i*th locus is present in open chromatin, and $\ell_{i}=0$ otherwise.

We impose a budget constraint “upper-bounding” the proportion of selected loci in a given input chromosome. Let b∈[0,1] be the maximum proportion of loci that can be selected, i.e.


$$\sum_{i=1}^{n}\ell_{i}\leq \left\lfloor nb\right\rfloor .$$

This constraint controls sensitivity in peak predictions and prevents unrealistic solutions in which an excessive fraction of chromatin is declared open. With estimates for the fraction of accessible chromatin hovering around 3%−4% of the human genome (Song *et al.* 2011, Sahinyan *et al.* 2022), we accordingly set *b* = 0.035 as the default value. Since the budget applies to each chromosome independently, and the accessibility for each chromosome can vary, the software implementation allows for chromosome-specific parameters to be defined.

To optimize selection of accessible regions, the following objective function is minimized:


(3) $$f(\ell )=\underset{\;\text{reward}\;\text{high}\;\text{locus}\;\text{scores}}{\underset{︸}{\sum_{i=1}^{n}-\left(S(i)\cdot \ell_{i}\right)}}+\underset{\gamma \;\text{controls}\;\text{influence}\;\text{of}\;\text{adjacent}\;\text{loci}}{\underset{︸}{\gamma \sum_{i=1}^{n-1}|\ell_{i}-\ell_{i+1}|}}.$$

**Figure 3.**
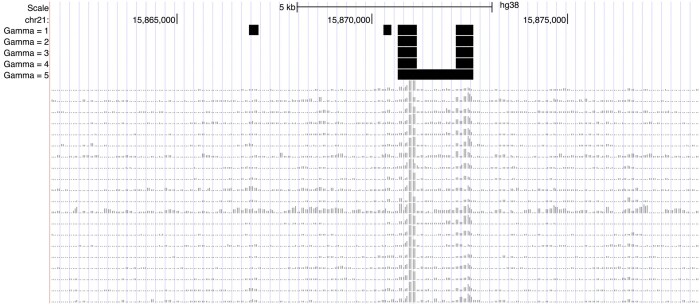
Example behavior of ROCCO in the UCSC Genome Browser (Karolchik *et al.* 2003) as *γ* is increased. The black bars in each track correspond to ROCCO’s predictions given the samples’ enrichment signals below. In the last row, we see that two distinct regions of enrichment are merged due to the strong influence of adjacent loci imposed by the *γ *= 5 parameter.

The first term rewards loci with high S scores, e.g. those with consistently high enrichment across samples or those on the edges of greatly enriched regions. The second term is introduced to account for spatial proximity of loci during optimization and controls the influence of signals in adjacent loci: for a given budget *b*, as *γ* is increased, fewer but longer distinct regions are annotated as open, yielding simpler solutions in a topological sense. This pattern is exhibited in Fig. 3.

To incorporate the described objective and budget constraint, we pose the following constrained optimization problem:


(4)  Minimize:ℓfIP(ℓ)=∑i=1n−(S(i)·ℓi)+γ∑i=1n−1|ℓi−ℓi+1| Subject To:(i)  ∑i=1nℓi≤⌊nb⌋(ii)  ℓi∈{0,1}, ∀i=1…n.

Constraint (ii) restricts the feasible region to integer solutions. In general, such constraints yield difficult optimization problems, e.g. because gradients are not defined for functions over the integers. Indeed, general integer programing is known to be NP-hard (Korte and Vygen 2012). A common remedy is to convert the original, integer-constrained formulation to an analogous problem with convenient analytic properties. Accordingly, we substitute the constraints


$$\ell_{i}\in \{0,1\}\to \;\ell_{i}\in R:0\leq \ell_{i}\leq 1$$

to obtain the following convex optimization problem:


(5)  Minimize:ℓfCP(ℓ)=∑i=1n−(S(i)·ℓi)+γ∑i=1n−1|ℓi−ℓi+1| Subject To:(i)  ∑i=1nℓi≤⌊nb⌋.(ii)  ℓi∈[0,1], ∀i=1…n.

As we will see, this formulation maintains the essence of the original problem in (4) and confers several useful properties. In general, convexity is a highly valued feature in constrained optimization as it ensures every local minimum is also a global minimum, thereby preventing instances of “premature” convergence to suboptimal solutions (Boyd and Vandenberghe 2004).

Theorem 1 *The problem in (5) can be solved in polynomial time for a globally optimal solution.*

Linear programs (LPs) are a special class of convex optimization problems in which both the objective and constraints are linear functions of the decision variables. Though general convex problems can often be solved efficiently in practice, there are certain intractable instances. In contrast, LPs can be solved in worst-case polynomial time with respect to the number of variables (Boyd and Vandenberghe 2004). Accordingly, the proof of Theorem 1, deferred to Supplementary Section S1.1), relies on showing that an optimal solution to (5), $\ell^{\;CP}\in R^{n}$, is obtained from the *n*-dimensional truncation of the optimal solution to the following LP:


(6)  Minimize:ℓfLP(ℓ)=∑i=1n−(S(i)·ℓi)+γ∑j=n+12n−1ℓj Subject To:(i)  ∑i=1nℓi≤⌊nb⌋(ii)  ℓi∈[0,1], ∀i=1…n(iii)  ℓj≥−1·(ℓi−ℓi+1), ∀i<n, j=n+i(iv)  ℓj≥+1·(ℓi−ℓi+1), ∀i<n, j=n+i,

which we denote as $\ell^{LP}\in R^{2n-1}$. Moreover, the optimal objective values for (5) and (6) are equal:


$$\min{\;f}_{\mathrm{CP}}=\min{\;f}_{\mathrm{LP}}=\mathrm{OPT}.$$

The time complexity of standard interior-point methods for solving LPs with *n* decision variables and a *d*-bit data representation is $O\left(n^{3}d\right)$, though several methods with improved worst-case bounds have been proposed (Karmarkar 1984, Vaidya 1989, den Hertog 1994). It is important to note that, in practice, modern solvers offer much greater efficiency than this worst-case bound might suggest by exploiting problem structure (Boyd and Vandenberghe 2004, Koch *et al.* 2022). Indeed, (6) possesses a particularly sparse objective and constraint matrix that allows for surprising efficiency showcased in Supplementary Material S1.3.

After solving the relaxed form of an integer program, it is often necessary to refine the solution for feasibility in the original integer-constrained region. Exact combinatorial techniques, such as branch and bound, can be applied to find optimal integer solutions; However, such methods may incur prohibitive computational expense and do not offer efficient runtime guarantees. It is often more practical to use an approximation scheme (Williamson and Shmoys 2011).

**Figure 4.**
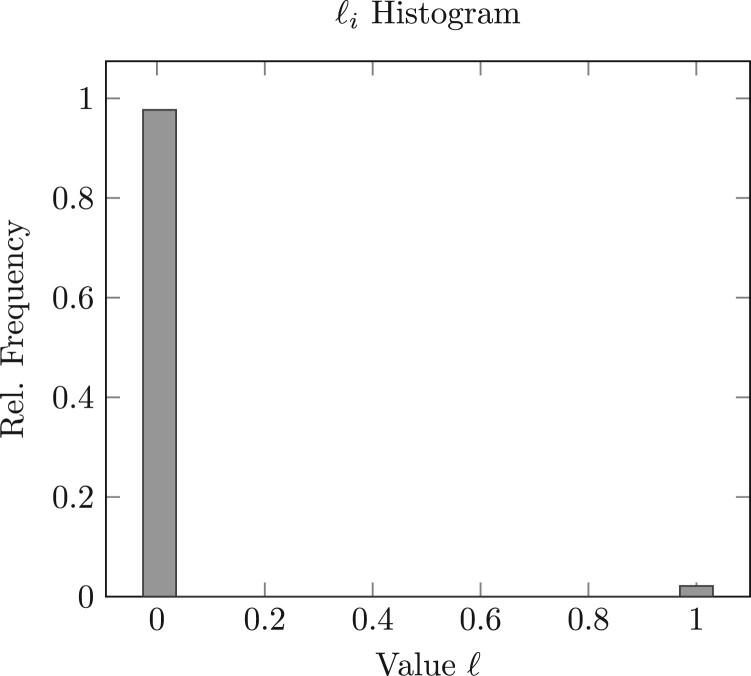
Observed distribution of decision variables after solving (6) with budgets b∈{.01,.025,.05,.075,.10} on 50 random subsamples (*K*=40) of the ATAC-seq data detailed in “Data availability” section and pooling the solutions from each run.


We find in our own experiments that solutions to (6) are nearly integral (Fig. 4) and can be rounded immediately, for instance, with the floor function, to obtain a feasible solution without a substantial sacrifice in practical performance. However, this near-integrality cannot be guaranteed in all experimental settings and parameter configurations, and a more robust procedure is preferred, which we now describe.

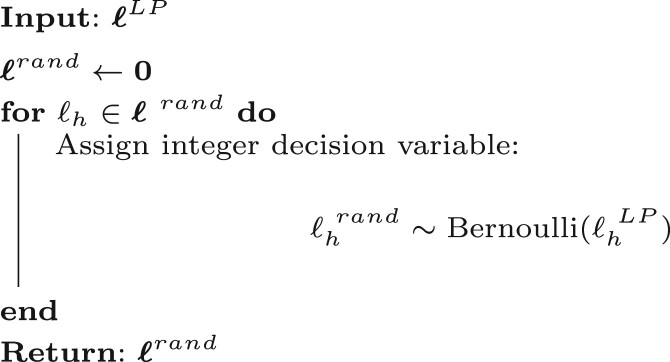
 
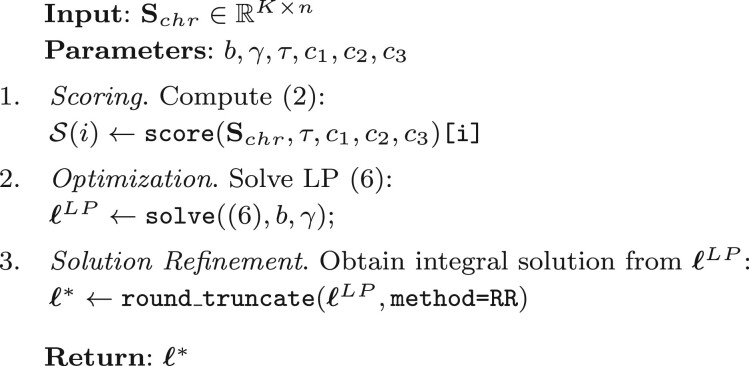


Given a solution to the relaxed formulation (6), we devise a procedure, denoted RR, based on randomized rounding (Raghavan and Tompson 1987), to obtain a set of candidate integral solutions, $L_{N}$. This set is generated by executing *N* iterations of Algorithm 1, after which RR picks the best feasible solution with the lowest objective value.

Note that in a given solution space $A\in {\mathbb{R}}^{D},D\geq 1\;$, integral solutions cannot yield better performance than the best real-valued solution. The set of integral solutions is a proper subset of A. For this reason, the quality of integer solutions can be judged with reference to $f_{\mathrm{LP}}(\ell^{LP})$. In light of this, the construction of solutions $\ell^{\;\mathrm{rand}}\in L_{N}$, with each $\ell_{i}^{\;\mathrm{rand}}$ defined as a Bernoulli-distributed random variable with parameter $p_{i}=\ell_{i}^{\;LP},$ grants convenient properties arising from linearity of expectation. Namely,


$$E\left[f_{\mathrm{LP}}(\ell^{\;\mathrm{rand}})\right]=\mathrm{OPT}$$

with constraints satisfied in expectation by $\ell^{\;\mathrm{rand}}$. We can use these expected values and leverage concentration inequalities to make probabilistic assertions regarding the solutions present in $L_{N}$.

Theorem 2 *Let* $L_{N}$*be a set of* N≥1*random solutions generated with Algorithm 1, and let c* *>* *1, a* *>* *0 be real numbers satisfying* $\frac{1}{c}+e^{-2n{\left(ab\right)}^{2}}<1$*for n loci and budget* b∈(0,1)*. Then with high probability*, $L_{N}$*contains at least one solution with both (i) an objective value no more than* c·OPT*and (ii) no more than* nb(1+a)*loci selected.*

In short, Theorem 2 is proven (Supplementary Material S1.2) using Markov’s and Hoeffding’s inequalities to show that the probability of one or more $\ell^{\mathrm{rand}}\in L_{N}$ satisfying both criteria is at least


$$1-{\left(\frac{1}{c}+e^{-2n{\left(ab\right)}^{2}}\right)}^{N},$$

which quickly approaches 1 for increasing *N*. We emphasize that this expression is a lower bound on the probability of a satisfying solution, and we often observed multiple such solutions in $L_{N}$ during the course of our experiments. But Theorem 2 allows us to make more general assertions under the supposition $\frac{1}{c}+e^{-2n{\left(ab\right)}^{2}}<1$. Since *n* is on the order of millions for default locus size *L*=50, this criterion is satisfied even for quite small c>1,a>0.

The RR procedure has linear time and space complexity, making it a minor contributor to overall computational expense. Though this random procedure technically renders ROCCO stochastic, for the default *N*=50 RR iterations, we observed only minor variation in solutions returned from independent runs of ROCCO. As seen in Supplementary Material S1.5, a pairwise Jaccard similarity matrix for five independently generated ROCCO solutions contains values no less than 0.9977.

Algorithm 2 offers a pseudocode representation of ROCCO as a whole.

Algorithm 1: Drawing *ℓ*^rand^

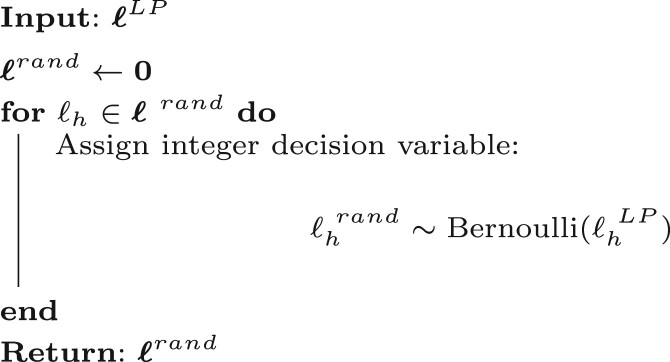

Algorithm 2: ROCCO

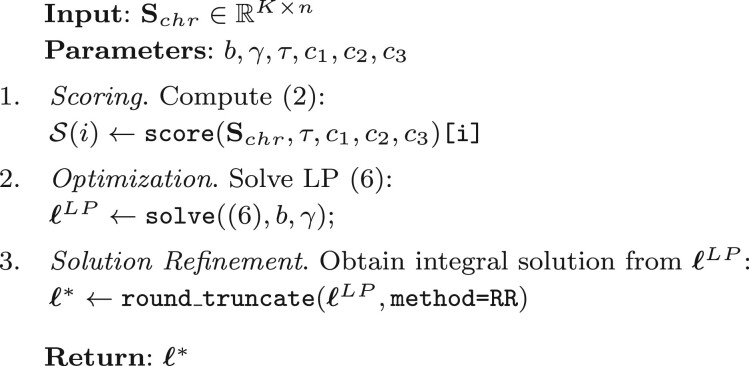

The object returned by Algorithm 2 is an *n*-dimensional decision vector, $\ell^{*},$ used to select loci as accessible. Note, contiguous selections (i.e. sequences of loci such that $\ell_{i}^{*}=1$) are merged into single peaks in the final BED file.

Remark 1 *Large Sample Sizes*. Note that only the scoring step is directly affected in runtime by the number of input samples *K*, running on the order of *nK* elementary operations using the median of medians algorithm. Practical scenarios satisfy K≪n, making the number of input samples a minor contributor to computational expense during the calculation of S(·). Further, because the scoring step is asymptotically dominated in worst-case computational expense by the optimization step, which is independent of *K*, the worst-case time complexity of ROCCO is likewise independent of the sample size, *K*.

## 3 Results

We performed several experiments to assess ROCCO’s detection performance using ATAC-seq data from 56 human lymphoblast samples generated within the ENCODE project (See "Data availability" section). Experiments were conducted using a stand-alone computer with an Intel Xeon CPU E5-2680 v3 @ 2.50 GHz processor, 8 cores, and 64 g RAM. We ran the utility, rocco prep, included in the ROCCO software distribution, to process BAM files and create enrichment signal tracks with L=50. The MOSEK solver (https://mosek.com), for which a free academic license can be readily obtained, is used to solve the linear program (LP) in (6). Note, ROCCO can call any open-source solver offered within the CVXPY (Diamond and Boyd 2016) platform, but runtimes may vary. ECOS (Domahidi *et al*. 2013)is a viable option installed with CVXPY by default. Additional analyses and details are available in the Supplementary Material.

### 3.1 Detection performance

A noteworthy limitation in experiments comparing performance of open chromatin detection methods is a lack of high-confidence annotations against which to test. However, to gauge performance and ensure viability, some proxy for ground truth is needed. Following Zhao and Boyle (2021), we constructed a “union set,” GT, of conservative irreproducible discovery rate-thresholded (Li *et al.* 2011) peaks from ENCODE “transcription factor” ChIP-seq experiments in the GM12878 lymphoblast cell line. We assume that the majority of annotated TF binding sites will correspond to open chromatin regions, but we note that variability in binding at a snapshot in time, the incomplete annotation of all TF binding, and cases where factors can bind to non-accessible chromatin introduce notable limitations. But, we argue that these data are sufficient to compare the relative performance of distinct methods. The $F_{\beta }$-score, defined below, was then used to assess the ability to recover and bound regions in GT using ATAC-seq data from the 56 independent samples. Details regarding the construction of the GT dataset can be found in Supplementary Material S1.7.

For each method, we generated consensus peaks using previously determined alignments for the *K*=56 samples. We then computed precision as


$$P=\frac{\left|D_{X}\cap D_{\mathrm{GT}}\right|}{\left|D_{X}\right|}$$

and recall as


$$R=\frac{\left|D_{X}\cap D_{\mathrm{GT}}\right|}{\left|D_{\mathrm{GT}}\right|},$$

where *D_X_* denotes the consensus peaks obtained from method *X* and set intersections in the numerators are computed using bedtools intersect (Quinlan and Hall 2010). The $F_{\beta }$-score was then calculated as the harmonic mean of precision and recall where recall is weighed *β* times as much as precision, i.e.


$$F_{\beta }=(1+\beta^{2})\frac{P\cdot R}{\beta^{2}P+R}.$$

As in Zhao and Boyle (2021), we use the $F_{\beta }$-score as the primary metric for comparison of methods since it intuitively combines both precision and recall and is less affected by extreme regions of the precision–recall curve that do not correspond to realistic use-cases.

#### 3.1.1 Detection performance: benchmark methods

Most methods to determine consensus peaks begin by identifying sample-specific peaks. For this step, we employed the widely used MACS2 software (Gaspar 2018) using parameters commonly specified for ATAC-seq experiments (see Section 5). With these, we used a common heuristic to specify consensus peaks (Yang *et al.* 2014). Namely, MACS2-Consensus only retained merged peaks supported by a majority of samples with a 100 bp tolerance in chromosome position across samples. Genrich is another method for consensus peak calling we tested that analyzes samples separately, calculating *P*-values for each. It then applies Fisher’s method to combine *P*-values at each genomic region. We also generated peak sets with MACS2-Pooled, which combined alignments from all samples into one BAM file and then used MACS2 to call peaks on this combined alignment file.

See Supplementary Material S1.8 for exact configurations used to produce results for these MACS2-based methods and for Genrich.

#### 3.1.2 Detection performance: results

**Figure 5.**
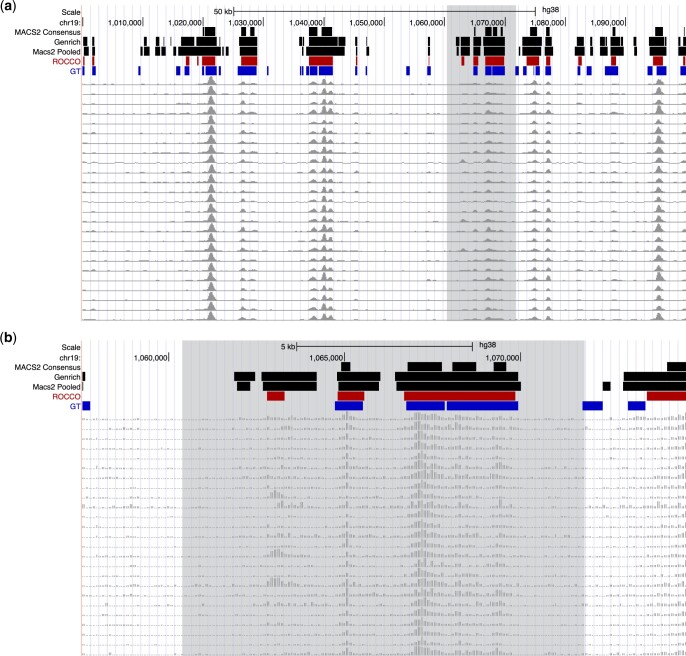
Example behavior over chr19:1000000–1100000. Consensus peak calls from each method tuned using the $F_{\beta }$ score (β=1.0). For perspective, results are displayed at two resolutions. (a) 100 kb and (b) 20 kb.

For an initial visual comparison of methods, Fig. 5 displays peak calls from each in 100 and 20 kb regions on Chromosome 19 in the UCSC Genome Browser (Karolchik *et al.* 2003). We also include ATAC-seq signals from 25 of the lymphoblast samples being evaluated. As expected, all methods identify regions with consistently strong signals across all samples. They vary, though, in the contiguity and boundaries of these regions. There are also method-specific regions, as well as ones called by multiple, but not all, methods.

To quantify genome-wide detection performance of the methods, we evaluated across several values


β∈{0.5,0.75,…,1.50,2.0}

to address a plausible but encompassing range of recall/precision prioritizations. The most extreme cases β=0.5,β=2.0 were included for completeness but may not be particularly well-motivated by realistic usage since the corresponding $F_{\beta }$ score can be unduly improved by simply rejecting any uncertain predictions or accepting all plausible predictions, respectively.

For each ${ℱ}_{\beta }$-score, we tuned each method over a range of significance thresholds deemed reasonable given their underlying models to maximize their performance. For Genrich, we tested


$$p\in \{10^{-6},10^{-5},\ldots ,10^{-1}\}.$$

For the MACS2-based methods, we tested:


q∈{.001,.005,.01,.05,.10,.20}.

For ROCCO, the budget parameter is most fundamental and upper-bounds the fraction of genomic region L that can be selected. We thus use *b* as the tuning parameter for ROCCO, leaving $\gamma ,\tau ,c_{1,2,3}$ as their default values, and evaluated:


b∈{.02,.025,…,.06}.

For each *β* listed above, given a method *X* and parameter *r*, we computed tuned performance as


$$\underset{r}{\max}F_{\beta }(X_{r}),$$

i.e. the best-observed performance of the method while sweeping its most fundamental parameter. These values are recorded in Table 2. ROCCO matched or exceeded the best performance of every benchmark method for all six *β* values. The performance disparity between ROCCO and the second best-scoring method was smallest for the most recall-dependent case *β *= 2, which we have stated is only partially informative and particularly vulnerable to spurious predictions.

**Table 2.** Performance for each method is recorded after tuning for the $F_{\beta }$ value in the leftmost column.

*β*	ROCCO	Genrich	MACS2-Pooled	MACS2-Consensus
0.50	**0.651**	0.460	0.390	0.643
0.75	**0.596**	0.489	0.427	0.586
1.0	**0.579**	0.520	0.465	0.545
1.25	**0.580**	0.545	0.501	0.517
1.50	**0.582**	0.566	0.531	0.498
2.0	**0.603**	0.595	0.577	0.475

Bold values indicate the greatest score in each row across the tested methods.

As mentioned above, ROCCO allows for specifying chromosome-specific parameters to account for varying chromatin state dynamics across the genome. For a cursory investigation into the effects of this practice, we tuned the budget parameter for $F_{1}$ via grid search for each chromosome. We observed a non-trivial increase in performance (${ℱ}_{1}=.620$) compared to a constant budget for all chromosomes (${ℱ}_{1}=.579$ as in Table 2), but we expect additional improvements from a more rigorous, technically sound approach in which budgets are not restricted to an arbitrary set of values.

A comparison of methods using their “default” significance thresholds without tuning is included in Supplementary Material S1.4, where ROCCO offers the greatest $F_{\beta }$-score in all but the β=0.5,β=2.0 experiments. Likewise, Supplementary Material S1.3 includes experiments assessing ROCCO’s computational efficiency in both theory and practice.

### 3.2 Variation in sample size/quality

Ideally, a consensus peak calling procedure will

- Effectively leverage data presented by multiple samples.
- Yield robust results in the presence of varying sample quality and size.
- Scale efficiently for large sample sizes.

In this section, we conduct several analyses to consider these aspects for ROCCO.

**Figure 6.**
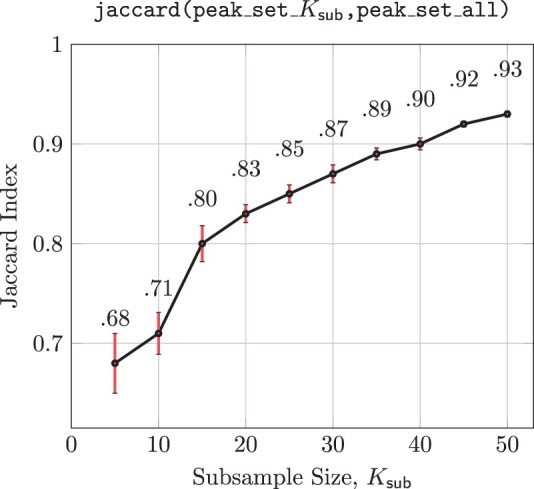
In 50 experiments for each $K_{\mathrm{sub}}\in \{5,10,15,\ldots ,50\},\;K_{\mathrm{sub}}$ ATAC-seq alignments are randomly subsampled and supplied as input to ROCCO. The 50 resulting output BED files are used to compute the average Jaccard index (95% CI) to ROCCO’s results obtained using all *K* = 56 samples.

In the first experiment, ROCCO is repeatedly executed using random subsamples of $K_{\mathrm{sub}}\in \{5,10,15,\ldots ,50\}$ ATAC-seq alignments from the dataset in “Data availability” section as input. The subsamples’ respective output peak sets are then compared to the peak set obtained by running ROCCO on the full set of *K*=56 ATAC-seq alignments. To compute similarity between the subsamples’ peak sets and the entire sample’s, we measured the Jaccard statistic between their respective BED files using bedtools (Quinlan and Hall 2010). In this context, the Jaccard index measures the ratio of the number of intersecting base pairs to the number of base pairs in the union of two BED files. The average Jaccard index for each $K_{\mathrm{sub}}$ is recorded in Fig. 6 along with 95% confidence intervals. Notably, with only $K_{\mathrm{sub}}=5$ samples, ROCCO generated peak sets roughly 70%—similar to the ROCCO’s peak set generated using all *K*=56 samples. Moreover, ROCCO produced strictly increasing Jaccard indexes for increasing subsample sizes, indicating an effective utilization of additional samples. Though the purpose of this experiment is to evaluate ROCCO’s approximation of the full-sample-derived results with respect to smaller subsamples, we note that detection performance as measured in Section 3.1 likewise improved with respect to increasing $K_{\mathrm{sub}}$ from $F_{1}=0.546\pm 0.011$ to $F_{1}=0.5783\pm .001$.

Regarding efficiency, the cpu-time required to execute ROCCO genome-wide was affected negligibly by $K_{\mathrm{sub}}$, with the average runtime for $K_{\mathrm{sub}}=5$ and $K_{\mathrm{sub}}=50$ differing by <20% despite the 1000% increase in samples. This result is informed theoretically by Remark 1, where the time complexity of ROCCO is shown to be asymptotically independent of sample size *K*.

**Figure 7.**
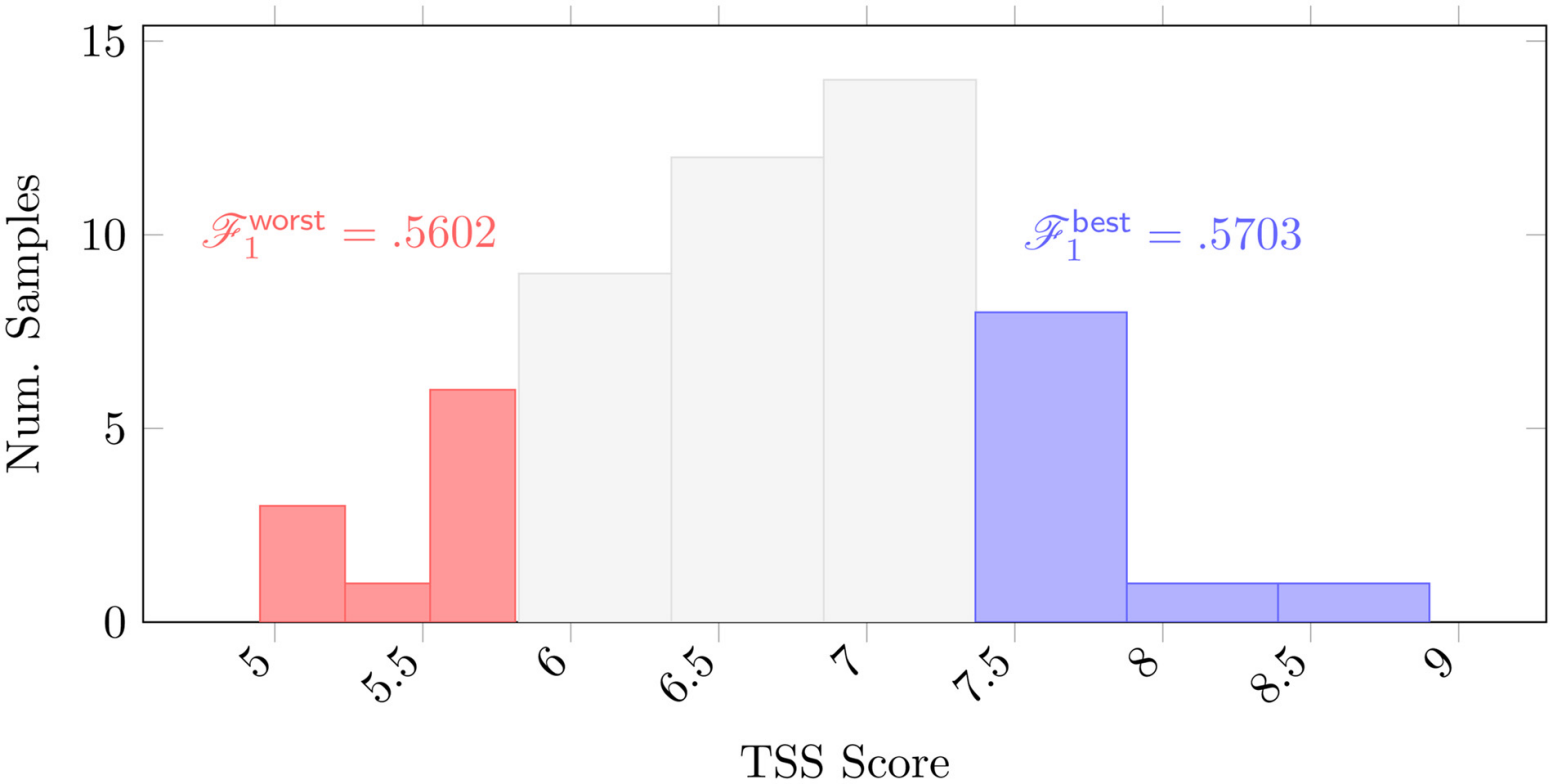
Histogram of TSS enrichment scores as defined by ENCODE for the *K* = 56 ENCODE ATAC-seq samples used in experiments. TSS scores are commonly used as a quality measure for ATAC-seq alignments. ROCCO was run twice with default parameters—once using the *K* = 10 “worst” samples (left/red) as input and again using the *K* = 10 “best” samples (right/blue). The $F_{1}$ performance in each case is labeled for comparison.

The second experiment compares the effect of data quality on consensus peak sets generated by executing ROCCO independently on the ten best and worst samples as measured by the transcription start site (TSS) enrichment score (Smith *et al.* 2021). The data from the 56 lymphoblast samples are of relatively good quality (Supplementary Material S1.6), as evidenced by minimum TSS enrichment score of 4.95. Nonetheless, the distribution of scores reflects appreciable differences in sample quality between the left and right tails. With this considered, the relatively small disparities in ROCCO’s detection performance shown in Fig. 7 indicate robustness to variation in sample quality. In comparison, MACS2-Consensus, the best-performing alternative method in this experimental setting, returns lower $F_{1}$-scores for both the worst 10 samples ($F_{1}=0.492$) and best 10 samples ($F_{1}=0.530$) and a larger disparity between performance in each case.

### 3.3 Differential accessibility testing with ROCCO

A key motivation in the development of ROCCO was for experimental designs where ATAC-seq data are generated from multiple samples within two or more distinct groups. Using these data, a key question regards the location of genomic regions over which accessibility differs significantly between groups. Knowledge of such regions may yield insights into regulatory mechanisms responsible for phenotypic differences. To offer a template for differential analysis with ROCCO, we ran a simple experiment comparing the accessibility landscapes of males and females in the lymphoblast data described in “Data availability” section. Note, a Jupyter Notebook tutorial addresses steps for differential analysis with ROCCO and is available on the GitHub repository.

In this demonstrative experiment, ROCCO was run independently on the 23 female and 33 male lymphoblast samples using default parameters, and the peaks were merged *post hoc* to create a final set of 172 933 consensus peaks, which we refer to as p-hoc_merge. p-hoc_merge included 23 865 peaks only detected in the male samples and 19 165 peaks only detected in the female samples. ∼17 000 peaks in each of these sets were not included in the set derived from running ROCCO on the input set of all *K*=56 samples, which we refer to as all_k. However, we note that the total span of disparate features was modest: a Jaccard similarity of ∼85% was observed between all_k and p-hoc_merge. Whether to split by group and then merge or to run ROCCO on all samples combined is a context-specific decision dependent on parity in sample sizes/quality among the cohorts and the general motivation of the experiment. We accommodate both protocols and have made each straightforward to apply in ROCCO’s software implementation.

Peak calling is an intermediate step in differential accessibility analyses to strategically identify candidate regions of interest, and DESeq2 (Love *et al.* 2014) was used in this experiment to detect significant differences in chromatin state between groups over the peak regions identified with ROCCO. At FDR-adjusted *P*<0.05, 3141 significant differentially accessible peaks spanning 2 275 100 bp were identified. About 93% (2916) of these peaks were observed in chromosome X, which is unsurprising given the recorded difference in sex between cohorts.

## 4 Discussion

In this manuscript, we introduced ROCCO, a novel method for identifying open chromatin regions in ATAC-seq data that simultaneously leverages information from multiple samples to determine a consensus set of peaks. ROCCO uses spatial features of enrichment signal data by initially formulating the problem with a convex model that can be solved with provable efficiency and performance guarantees. Importantly, the model accounts for features common to the edges of accessible chromatin regions, which are often hard to determine based on independently determined sample peaks that can vary widely in their genomic locations. In addition to several attractive conceptual and theoretical features, ROCCO also exhibited improved detection performance based on ATAC-seq data from 56 lymphoblast samples evaluated against known TF binding sites determined using ChIP-seq. ROCCO is especially suited for experimental designs that include multiple samples from two or more distinct groups with one goal being to determine regions that are differentially accessible between these groups. A Jupyter notebook tutorial provides a step-by-step protocol for this with all necessary scripts provided on the GitHub repository.

For simplicity and to provide a conservative comparison with other methods, we ran ROCCO with the same genome-wide parameters for all chromosomes, including the budget which dictates the “maximum” proportion of the chromosome that should be considered accessible. However, chromatin accessibility varies across chromosomes, and ROCCO’s performance may be improved by exploiting properties specific to each chromosome. We found that optimizing the budget parameter for each chromosome for $F_{\beta }$-score at β=1.0 did show an improvement. These optimized budget parameters roughly reflected the differences in gene density and read density across chromosomes, as expected. In the future, we will focus on developing an efficient, robust method to derive reasonable chromosome-specific budgets based on the input signal data.

ROCCO’s locus size parameter was set to *L*=50 throughout experiments. While our results suggest this grants good performance and a resolution sufficient to identify both broad and concentrated regions of enrichment, it may prove beneficial to modify this parameter depending on the expected size of elements and desired granularity. We note, however, that decreasing *L* increases the number of loci, *n*, which may induce additional computational expense. By the same reasoning, computational burden can be reduced by increasing *L*, though some loss in the precision of predicted peaks may result. The effects of the locus size parameter are discussed in greater detail in Supplementary Material S1.4.

Overall, ROCCO represents a scalable, effective, and mathematically sound method that is broadly applicable and addresses an important need in functional genomics analysis.

## Supplementary Material

btad725_Supplementary_Data

## Data Availability

The ATAC-seq data from 56 lymphoblast samples used to conduct experiments were obtained from the ENCODE Project (Luo *et al.* 2019). Specifically, we used alignments that had been determined according to the ENCODE ATAC-seq protocol. Note, we remove chromosome Y from consideration to ensure each sample contained data for the same set of chromosomes. A link to the metadata with accession codes for this dataset is available in Supplementary Material S1.6.

## References

[btad725-B1] Bao X , Rubin AJ, Qu K et al A novel ATAC-seq approach reveals lineage-specific reinforcement of the open chromatin landscape via cooperation between BAF and p63. Genome Biol 2015;16:284. 26683334 10.1186/s13059-015-0840-9 PMC4699366

[btad725-B2] Bentsen M , Goymann P, Schultheis H et al ATAC-seq footprinting unravels kinetics of transcription factor binding during zygotic genome activation. Nat Commun 2020;11:4267. 32848148 10.1038/s41467-020-18035-1 PMC7449963

[btad725-B3] Boyd S , Vandenberghe L. Convex Optimization. Cambridge, UK: Cambridge University Press, 2004.

[btad725-B4] Boyle AP , Davis S, Shulha HP et al High-resolution mapping and characterization of open chromatin across the genome. Cell 2008;132:311–22. 18243105 10.1016/j.cell.2007.12.014 PMC2669738

[btad725-B5] Buenrostro JD , Wu B, Chang HY et al ATAC-seq: a method for assaying chromatin accessibility genome-wide. Curr Protoc Mol Biol 2015;109:21.29.1–9. 10.1002/0471142727.mb2129s109 PMC4374986 25559105

[btad725-B6] Corces MR , Granja JM, Shams S et al; Cancer Genome Atlas Analysis Network. The chromatin accessibility landscape of primary human cancers. Science 2018;362:eaav1898. 30361341 10.1126/science.aav1898 PMC6408149

[btad725-B7] den Hertog D. Interior Point Approach to Linear, Quadratic and Convex Programming. Dordrecht, NL: Springer Netherlands, 1994.

[btad725-B8] Diamond S , Boyd S. CVXPY: a python-embedded modeling language for convex optimization. J Mach Learn Res 2016;17:1–5. PMC4927437 27375369

[btad725-B9] Domahidi A , Chu E, Boyd S. ECOS: an SOCP solver for embedded systems. In: *European Control Conference (ECC)*, Zurich, Switzerland. 3071–6. IEEE, 2013.

[btad725-B10] Fisher R. Statistical Methods for Research Workers. Edinburgh, UK: Oliver & Boyd, 1925.

[btad725-B11] Gaspar JM. Improved peak-calling with MACS2. bioRxiv, 2018, preprint: not peer reviewed.

[btad725-B12] Guerin LN , Barnett KR, Hodges E. Dual detection of chromatin accessibility and DNA methylation using ATAC-me. Nat Protoc 2021;16:5377–97. 34663963 10.1038/s41596-021-00608-z PMC11057009

[btad725-B13] Hofvander J , Puls F, Pillay N et al Undifferentiated pleomorphic sarcomas with PRDM10 fusions have a distinct gene expression profile. J Pathol 2019;249:425–34. 31313299 10.1002/path.5326

[btad725-B14] Karmarkar N. A new polynomial-time algorithm for linear programming. In: *Proceedings of the Sixteenth Annual ACM Symposium on Theory of Computing*, STOC ’84, Washington DC, USA. 302–11. New York, NY: Association for Computing Machinery, 1984.

[btad725-B15] Karolchik D , Baertsch R, Diekhans M et al; University of California Santa Cruz. The UCSE Genome Browser Database. Nucleic Acids Res 2003;31:51–4. 12519945 10.1093/nar/gkg129 PMC165576

[btad725-B16] Koch T , Berthold T, Pedersen J et al Progress in mathematical programming solvers from 2001 to 2020. EURO J Comput Optim 2022;10:100031.

[btad725-B17] Korte B , Vygen J. Combinatorial Optimization: Theory and Algorithms. 5th edn. New York, New York: Springer Publishing Company, Incorporated, 2012.

[btad725-B18] Li G , Reinberg D. Chromatin higher-order structures and gene regulation. Curr Opin Genet Dev 2011;21:175–86. 21342762 10.1016/j.gde.2011.01.022 PMC3124554

[btad725-B19] Li Q , Brown JB, Huang H et al Measuring reproducibility of high-throughput experiments. Ann Appl Stat 2011;5:1752–79.

[btad725-B20] Love MI , Huber W, Anders S. Moderated estimation of fold change and dispersion for RNA-seq data with DESeq2. Genome Biol 2014;15:550. 25516281 10.1186/s13059-014-0550-8 PMC4302049

[btad725-B21] Luo Y , Hitz BC, Gabdank I et al New developments on the encyclopedia of DNA elements (ENCODE) data portal. Nucleic Acids Res 2019;48:D882–9. 10.1093/nar/gkz1062 PMC7061942 31713622

[btad725-B22] Ming H , Sun J, Pasquariello R et al The landscape of accessible chromatin in bovine oocytes and early embryos. Epigenetics 2021;16:300–12. 32663104 10.1080/15592294.2020.1795602 PMC7901547

[btad725-B23] Pham-Gia T , Hung T. The mean and median absolute deviations. Math Comput Model 2001;34:921–36.

[btad725-B24] Quinlan AR , Hall IM. BEDTools: a flexible suite of utilities for comparing genomic features. Bioinformatics 2010;26:841–2. 20110278 10.1093/bioinformatics/btq033 PMC2832824

[btad725-B25] Raghavan P , Tompson CD. Randomized rounding: a technique for provably good algorithms and algorithmic proofs. Combinatorica 1987;7:365–74.

[btad725-B26] Roy A , Harrar SW, Konietschke F. The nonparametric Behrens-Fisher problem with dependent replicates. Stat Med 2019;38:4939–62. 31424122 10.1002/sim.8343

[btad725-B27] Sahinyan K , Blackburn DM, Simon M-M et al Application of ATAC-Seq for genome-wide analysis of the chromatin state at single myofiber resolution. eLife 2022;11:e72792. 35188098 10.7554/eLife.72792 PMC8901173

[btad725-B28] Salavati M , Woolley SA, Araya YC et al Profiling of open chromatin in developing pig (*Sus scrofa*) muscle to identify regulatory regions. G3 (Bethesda) 2021;12:jkab424. 10.1093/g3journal/jkab424 PMC9210303 34897420

[btad725-B29] Smith JP , Corces MR, Xu J et al PEPATAC: an optimized pipeline for ATAC-seq data analysis with serial alignments. NAR Genom Bioinform 2021;3:lqab101. 34859208 10.1093/nargab/lqab101 PMC8632735

[btad725-B30] Song L , Zhang Z, Grasfeder LL et al Open chromatin defined by DNaseI and FAIRE identifies regulatory elements that shape cell-type identity. Genome Res 2011;21:1757–67. 21750106 10.1101/gr.121541.111 PMC3202292

[btad725-B31] Tsaryk R , Yucel N, Leonard EV et al Shear stress switches the association of endothelial enhancers from ETV/ETS to KLF transcription factor binding sites. Sci Rep 2022;12:4795. 35314737 10.1038/s41598-022-08645-8 PMC8938417

[btad725-B32] Vaidya P. Speeding-up linear programming using fast matrix multiplication. In: *30th Annual Symposium on Foundations of Computer Science*, Durham, NC. IEEE, 1989.

[btad725-B33] Wang J , Zibetti C, Shang P et al ATAC-seq analysis reveals a widespread decrease of chromatin accessibility in age-related macular degeneration. Nat Commun 2018;9:1364. 29636475 10.1038/s41467-018-03856-y PMC5893535

[btad725-B34] Williamson DP , Shmoys DB. The Design of Approximation Algorithms. Cambridge, UK: Cambridge University Press, 2011.

[btad725-B35] Yang Y , Fear J, Hu J et al Leveraging biological replicates to improve analysis in ChIP-seq experiments. Comput Struct Biotechnol J 2014;9:e201401002. 24688750 10.5936/csbj.201401002 PMC3962196

[btad725-B36] Zhao N , Boyle AP. F-Seq2: improving the feature density based peak caller with dynamic statistics. NAR Genom Bioinform 2021;3:lqab012. 33655209 10.1093/nargab/lqab012 PMC7902237

