# Supplementary material for: ROCCO: a robust method for detection of open chromatin via convex optimization
Source: Bioinformatics. 2023 Nov 29;39(12):btad725. doi: 10.1093/bioinformatics/btad725 (PMC10715771; doi:10.1093/bioinformatics/btad725)
Supplement: btad725_Supplementary_Data [file btad725_supplementary_data.pdf]

1. Supplementary Material

1.1. Proof of Theorem I

For easy reference, the optimization problem (5) is restated here.

$$\begin{aligned} \underset{\boldsymbol{\ell}}{\text{Minimize:}} \quad & f_{\text{CP}}(\boldsymbol{\ell}) = \sum_{i=1}^{n} -\left(\mathcal{S}(i)\cdot \ell_i\right) + \gamma \sum_{i=1}^{n-1} |\ell_i - \ell_{i+1}| \\ \text{Subject To:} \quad & \text{(i)} \ \sum_{i=1}^{n} \ell_i \leq \lfloor nb \rfloor. \\ & \text{(ii)} \ \ell_i \in [0,1], \ \forall i = 1\ldots n. \end{aligned}$$

Theorem 1 *(5) can be solved in polynomial time for a globally optimal solution.*

Proof The crux of the proof is to show that (5) can be expressed as a linear program (LP): That is, a constrained optimization problem with real-valued decision variables in which the objective function and constraints are linear. These conditions maintain a convex feasible region of solutions with a unique (global) minimum value. Efficiency then follows from the use of interior-point algorithms with well-known polynomial time complexities for LPs (den Hertog, 1994).

A quick remedy for modelling the non-linearity of f is to introduce additional variables, $\ell_j : \ \forall \ i < n, j = n+i$ to replace the absolute value functions in the objective

$$f_{\text{LP}}(\boldsymbol{\ell}) = \sum_{i=1}^{n} -\left(\mathcal{S}(i)\cdot \ell_i\right) + \gamma \sum_{j=n+1}^{2n-1} \ell_j$$

and add constraints such that:

$$\ell_j \geq -(\ell_i - \ell_{i+1}), \ \ell_j \geq (\ell_i - \ell_{i+1}).$$

Since we are *minimizing* f, in any optimal solution, the above constraints imply

$$\ell_j = \max\{\ell_i - \ell_{i+1}, \ell_{i+1} - \ell_i\} = |\ell_i - \ell_{i+1}|$$

for all ℓ_i. Thus, the truncated n-dimensional solution to the following LP:

$$\begin{aligned} \underset{\boldsymbol{\ell}}{\text{Minimize:}} \quad & f_{\text{LP}}(\boldsymbol{\ell}) = \sum_{i=1}^{n} -\left(\mathcal{S}(i)\cdot \ell_i\right) + \gamma \sum_{j=n+i}^{2n-1} \ell_j \\ \text{Subject To:} \quad & \text{(i)} \ \sum_{i=1}^{n} \ell_i \leq \lfloor nb \rfloor \\ & \text{(ii)} \ \ell_i \in [0,1], \ \forall i = 1\ldots n \\ & \text{(iii)} \ \ell_j \geq -1\cdot(\ell_i - \ell_{i+1}), \ \forall i < n, \ j = n+i \\ & \text{(iv)} \ \ell_j \geq +1\cdot(\ell_i - \ell_{i+1}), \ \forall i < n, \ j = n+i \end{aligned}$$

is an optimal solution for (5), which is what we needed to show. □

1.2. Proof of Theorem 2

The following two results are are useful in proving Theorem 2.

Lemma 1 *Let $\boldsymbol{\ell}^{\ rand}$ be a solution generated with the procedure in Algorithm 1 and let $\boldsymbol{\ell}^{LP}$ be an optimal solution to 6. Then $\boldsymbol{\ell}^{\ rand}$ is integral and possesses the following properties*

1. *The objective value of $\boldsymbol{\ell}^{\ rand}$ is equal in expectation to the objective value of $\boldsymbol{\ell}^{\ LP}$:*

$$\mathbb{E}[f_{\text{LP}}(\boldsymbol{\ell}^{\ rand})] = f_{\text{LP}}(\boldsymbol{\ell}^{\ LP}) = \mathsf{OPT}$$

2. *The randomized solution satisfies the budget in expectation:*

$$\mathbb{E}\left[\sum_{i=1}^{n} {\ell_i}^{rand}\right] \leq \lfloor nb \rfloor$$

Both of the statements above follow as consequences of linearity of expectation.

Note, without loss of generality, we can treat the objective function f as non-negative in order to satisfy the condition for Markov's inequality, used in proof of Theorem 2. Because f is bounded below, we can always add a constant to ensure $\min f \geq 0$ without affecting optimality of solutions since

$$\arg\min_{\boldsymbol{\ell}} \; f(\boldsymbol{\ell}) = \arg\min_{\boldsymbol{\ell}} \; f(\boldsymbol{\ell}) + C.$$

Theorem 2 *Let $\mathbf{L}_N$ be a set of $N \geq 1$ random solutions generated with Algorithm 1, and let $c > 1$, $a > 0$ be real numbers satisfying $\frac{1}{c} + e^{-2n(ab)^2} < 1$ for n loci and budget $b \in (0,1)$. Then with high probability, $\mathbf{L}_N$ contains at least one solution with both (a) an objective value no more than $c \cdot \mathsf{OPT}$ and (b) no more than $nb(1+a)$ loci selected.*

Proof To prove the result, we find a lower bound for the probability that these two criteria occur simultaneously for some solution in $\mathbf{L}_N$, and that this probability approaches 1 as $N \to \infty$. Note, for concision, we let $S_n = \sum_{i=1}^{i=n} \ell_i^{rand}$.
Now, using the expected value for the objective given in Lemma 1, applying Markov's inequality yields:

$$\mathbb{P}\left[\underbrace{f_{\text{LP}}(\boldsymbol{\ell}^{\,rand}) \geq c \cdot f_{\text{LP}}(\boldsymbol{\ell}^{LP})}_{\text{Event A}}\right] \leq \frac{1}{c}.$$

Likewise, recalling that each ℓ_i^{rand} is generated as an independent random variable, we can apply Hoeffding's inequality and Lemma 1 to obtain:

$$\mathbb{P}\left[\underbrace{S_n \geq (1+a) \cdot nb}_{\text{Event B}}\right] \leq e^{-2n(ab)^2}.$$

Combining these two (possibly dependent) events with the union bound, we get

$$\mathbb{P}\left[A \cup B\right] \leq \mathbb{P}[A] + \mathbb{P}[B] \leq \frac{1}{c} + e^{-2n(ab)^2}.$$

By construction, each solution in $\mathbf{L}_N$ is independent and identically distributed. Since the joint probability of N independent events $\{E_1, E_2, \ldots, E_N\}$, each satisfying $\mathbb{P}[E_i] \leq m_p$, is no more than $(m_p)^N$, we can obtain an upper bound for the probability that at least one of the events, A or B, occurs in *every* solution in $\mathbf{L}_N$:

$$\mathbb{P}\left[\underbrace{\forall\, \boldsymbol{\ell}^{\,rand} \in \mathbf{L}_N : \; A \cup B}_{\text{Event C}}\right] \leq \left(\frac{1}{c} + e^{-2n(ab)^2}\right)^N$$

We are interested in the event that,

$$\exists\, \boldsymbol{\ell}^{\,rand} \in \mathbf{L}_N : \; \neg(A \cup B),$$

or, equivalently,

$$\exists\, \boldsymbol{\ell}^{\,rand} \in \mathbf{L}_N : \; \neg A \;\cap\; \neg B,$$

where at least one solution exists in $\mathbf{L}_N$ such that neither A nor B occur. This is the complement of Event C and occurs with probability at least

$$1 - \left(\frac{1}{c} + e^{-2n(ab)^2}\right)^N.$$

More explicitly, we have:

$$\mathbb{P}\left[\exists\, \boldsymbol{\ell}^{\,rand} \in \mathbf{L}_N : \; f_{\text{LP}}(\boldsymbol{\ell}^{\,rand}) < c \cdot f_{\text{LP}}(\boldsymbol{\ell}^{\,LP}),\; S_n < nb(1+a)\right] \geq 1 - \left(\frac{1}{c} + e^{-2n(ab)^2}\right)^N$$

which approaches 1 for increasing N. □

1.3. Computational Efficiency

In this section, we discuss `ROCCO`'s computational efficiency from both theoretical and practical perspectives. Unless otherwise stated, `ROCCO`'s default parameters and the computing environment discussed in Section 5.1 are used.

The worst-case time complexity of `ROCCO` is equivalent to the worst-case time complexity required to solve a linear program (LP) with n variables and a d-bit data representation, since this step dominates the others asymptotically (See Algorithm 2). However, the $\mathcal{O}(n^3 d)$ bound of standard interior-point solvers is of little insight for the vast majority of practical problems using current methodology and is only realized in pathological cases with completely dense matrices. A detailed survey of the improved capabilities of modern solvers and

the multitude of algorithmic and hardware-based advances experienced in the past two decades is out of scope for this paper, but readers are referred to Koch et al. (2022).

To gain a practical sense of `ROCCO`'s computational expense, an empirical analysis is warranted. To this end, we ran `ROCCO` genome-wide in 50 independent trials on the data set described in Section 5.2. The observed mean runtime and maximum resident set size (MRSS) for each chromosome were then plotted, with the 95%-confidence intervals[4] thereof shaded to account for uncertainty arising from stochastic factors at the operating system and processor-level in Figure S1.

Table S1. Chromosome Sizes (bp) in `hg38`

Chromosome	Base pairs (Millions)
1	248.96
2	242.19
3	198.30
4	190.21
5	181.54
6	170.81
7	159.35
8	145.14
9	138.39
10	133.80
11	135.09
12	133.28
13	114.36
14	107.04
15	101.99
16	90.34
17	81.28
18	78.08
19	59.13
20	63.03
21	48.13
22	50.96
X	155.27
Y	59.37

Chromosome size proved to be a fundamental contributor to computational expense. For example, `chr1`—the largest of human chromosomes—yielded the greatest average runtime and MRSS. In contrast, the smallest chromosome, `chr21`, required roughly one-tenth the time of `chr1`. See Table S1 for the number of base pairs in each human chromosome, scaled to millions.

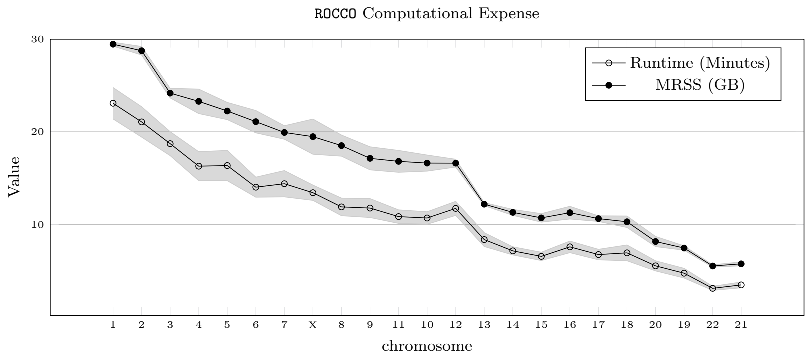

Fig. S1: Average runtime and peak memory (MRSS) expended by `ROCCO` for each chromosome.

We note that `ROCCO`'s design allows for straightforward parallelization, since each chromosome can be processed independently. An option for simultaneous execution of `ROCCO` on multiple chromosomes is available in the software implementation via `rocco gwide --multi`. This feature can dramatically reduce the runtime of `ROCCO` if multiple cores are available. However, to accommodate users in limited

[4] The confidence intervals were computed according to a t-distribution with $\nu = 49$.

computing environments, the default behavior of `rocco gwide` is to run the method sequentially on each chromosome, leading to an average genome-wide runtime of 4.3 hours in this experiment[5].

1.4. ROCCO Parameters

ROCCO is designed to require minimal fine-tuning. Many of the parameters are included only for completeness and can be ignored for common use-cases. The default parameters provide generally strong performance as shown in Table S2, but we highlight several potentially consequential dynamics for users wishing to configure ROCCO optimally for a particular setting. In particular, the budget parameter may non-trivially affect behavior of the method.

1.4.1. Constant Budgets

The budget, b, is a fundamental parameter of ROCCO as it determines an *upper-bound* on the proportion of loci that can be selected. A genome-wide, constant budget can yield strong detection performance as demonstrated in Tables 2, S2. The best constant budget depends on users' preferences regarding recall/precision, as seen in Figure S2. Intuitively, for increasing β, that is, an increased emphasis on recall than precision, the best-scoring budget b is increasing.

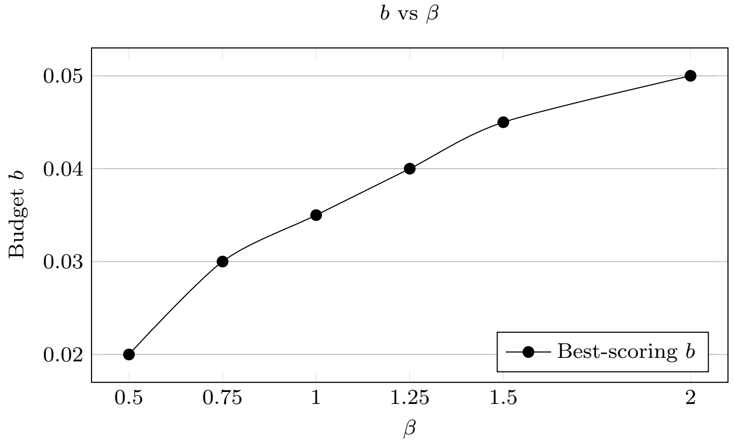

Fig. S2: As greater importance is placed on recall than precision (β is increased), the $\mathcal{F}_\beta$-tuned budget increases.

1.4.2. Chromosome-Specific Budgets

A notion of dynamic, region-specific budgets is naturally appealing given chromatin's variable dynamics throughout the genome. ROCCO's software implementation allows users to specify budgets (and other parameters as well) at the chromosome level using `rocco gwide`'s `-p/--param_file` parameter. See `hg38_params.csv` for suggested budgets for each chromosome in the `hg38` assembly. Using these chromosome-specific budgets to run ROCCO genome-wide on the ATAC-seq data in Section 5.2, $\mathcal{F}_1 = .592$ was obtained as compared to $\mathcal{F}_1 = .579$ with an invariant, genome-wide budget (Table 2).

In the software implementation, `rocco budgets` can also be used to compute chromosome-specific budgets. In this procedure, estimates for the relative read densities of each chromosome are scaled such that they average to a user-specified value, e.g., $b = .035$. The ranking of chromosomes' budgets according to read density is preserved under this operation, and the scaling step produces interpretable budget values as we have defined them. See the API reference and corresponding script for more details.

1.4.3. Locus Size, L and γ

At higher resolutions, it is natural to expect less variation between proximal elements, as the absolute distance between them is lesser by definition. Consequently, for smaller locus sizes L, users may wish to enforce a stronger dependence between adjacent loci by increasing γ. As a brief demonstration in Figure S3, all parameters except γ, L are fixed at their defaults, and we observe greater fragmentation in ROCCO's solution with ($\gamma = 1, L = 25$) than the ($\gamma = 2, L = 25$), ($\gamma = 1, L = 50$), and ($\gamma = 2, L = 50$) cases.

[5] In a personal computing environment, `rocco gwide` successfully returned genome-wide results on a MacBook (Model A2485) in under five hours.

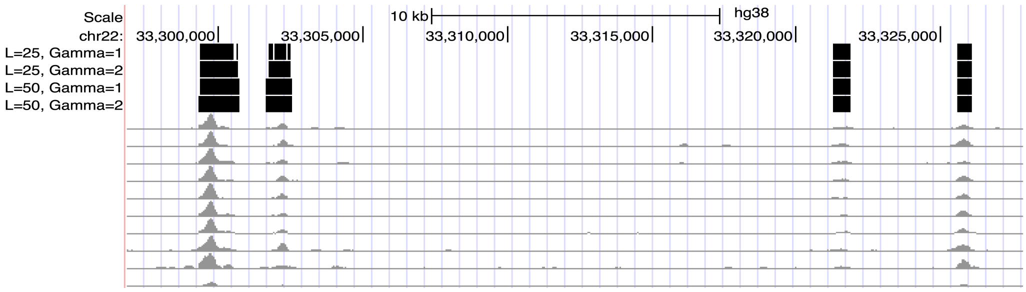

Fig. S3: *Relationship between γ and L.* `ROCCO`'s annotations on a demonstrative 10kb subset of chromosome 22 are displayed. `ROCCO` is run four times on a set of ten ATAC-seq alignments with all parameters default/fixed except γ, L.

1.4.4. Default Performance

Genome-Wide Detection Performance—No Tuning

β	`ROCCO`	`Genrich`	`MACS2-Pooled`	`MACS2-Consensus`
0.50	0.597	0.277	0.329	**0.642**
0.75	**0.587**	0.317	0.369	0.565
1.0	**0.579**	0.363	0.413	0.511
1.25	**0.572**	0.410	0.455	0.501
1.50	**0.568**	0.454	0.493	0.452
2.0	0.562	0.530	**0.565**	0.424

Table S2. $\mathcal{F}_\beta$-score obtained from each method using their default significance threshold.

To measure "out-of-box" performance of `ROCCO`, we also compare each method using their *default* significance thresholds: $b = .035$, $q = .05$, $p = .01$ for `ROCCO`, MACS2, and Genrich, respectively. In Table S2, we see that `ROCCO` outperforms the benchmarks in four of six experiments. The two experiments in which `ROCCO` does not achieve best performance are for the minimum and maximum β values, where recall is weighed half and twice as much as precision, respectively. Users with a strong preference for either recall or precision are advised to tune accordingly.

1.5. Randomness of `RR` Procedure

Using the data set described in Section 5.2, `ROCCO` is run five times independently using the default $N = 50$ `RR` iterations. The pairwise Jaccard statistics between resulting peak sets are then computed in Table S3.

$\mathcal{J}$	$\boldsymbol{\ell}_1^*$	$\boldsymbol{\ell}_2^*$	$\boldsymbol{\ell}_3^*$	$\boldsymbol{\ell}_4^*$	$\boldsymbol{\ell}_5^*$
$\boldsymbol{\ell}_1^*$	1	0.997758	0.997745	0.997765	0.997743
$\boldsymbol{\ell}_2^*$	.	1	0.997734	0.997745	0.997714
$\boldsymbol{\ell}_3^*$	.	.	1	0.997721	0.997731
$\boldsymbol{\ell}_4^*$	.	.	.	1	0.997738
$\boldsymbol{\ell}_5^*$	.	.	.	.	1

Table S3. Pairwise Jaccard indexes of peak sets obtained during five separate genome-wide runs of `ROCCO` with default parameters and $N = 50$ `RR` iterations.

1.6. ATAC-seq Data

The 56 ATAC-seq samples used for experiments in this paper are available from the ENCODE Project. These BAM files result from ENCODE's standard ATAC-seq protocol
`https://www.encodeproject.org/pipelines/ENCPL344QWT/`.

Download metadata for ATAC-seq samples:
`https://www.encodeproject.org/metadata/?control_type%21=%2A&status=released&perturbed=false&assay_title=ATAC-seq&biosample_ontology.cell_slims=lymphoblast&files.file_type=bam&type=Experiment`

We select the BAM files corresponding to rows with `Output Type='alignments'`.

1.7. Conservative IDRt TF Chip-Seq Union Peaks

Available from:
`https://github.com/Boyle-Lab/F-Seq2-Paper-Supplementary/blob/d9c5357e615535240b09fac6567874bf626b7652/ATAC-seq%20benchmarking/data_process/download_preprocess_data.ipynb`

We then used the texttttliftOver UCSC binary tool `genome.ucsc.edu/cgi-bin/hgLiftOver` to convert to `hg38`.

1.8. Alternative Methods - Configurations

1.8.1. MACS2-Based Methods

The following configuration of `MACS2` is used to call peaks in each sample for each significance threshold.

```
macs2 callpeak -t {BAM file} -f BAMPE  --nomodel  --nolambda \
--keep-dup all -q {significance threshold}
```

1.8.2. Genrich

Genrich is called with the following configuration for each significance threshold

```
./Genrich -t {comma-separated list of name-sorted BAM files} -j \
-p {significance threshold}
```

The `-j` option specifies ATAC-seq mode

1.9. Peak Width Distributions

Each method is tuned for the $\mathcal{F}_1$-score as in Table 2 Row 3, and their respective peak-width distributions plotted in Figure S4. For each method, there is a consistent majority of peaks less than 500 bps with strictly decreasing frequency past this length.

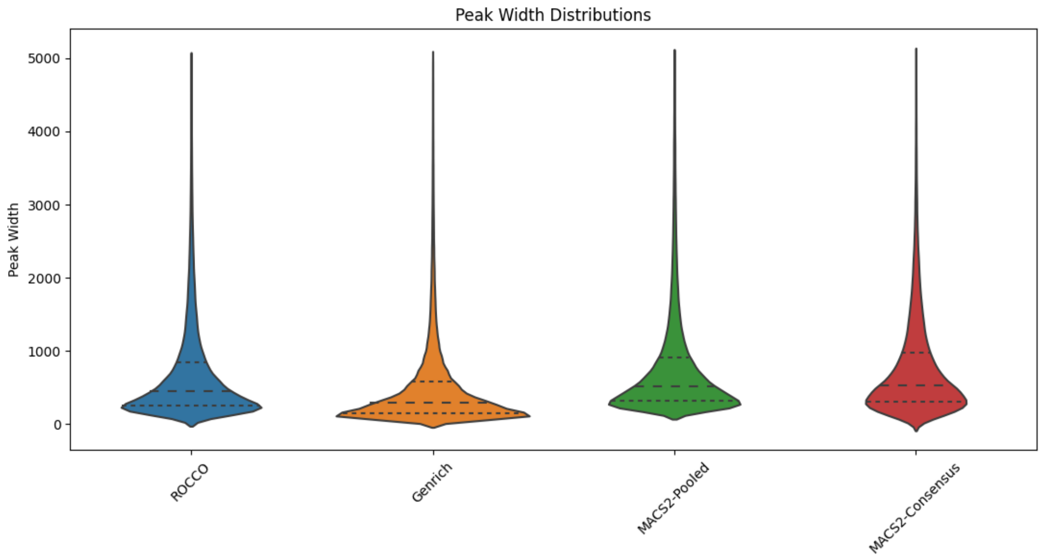

Fig. S4: Violin plots of the peak width distributions for each method.
